# Outcomes of modern conservative burn care in children treated outside a burn center: a four-year retrospective analysis of 520 patients

**DOI:** 10.1186/s12887-026-06522-1

**Published:** 2026-01-28

**Authors:** Volkan Altınok, Onur Yalçın, Aybegüm Kalyoncu Aycenk, Ecem İpek Altınok

**Affiliations:** 1 Department of Pediatric Surgery, Faculty of Medicine, Ordu University, Ordu, Türkiye; 2 Department of Pediatrics, Faculty of Medicine, Ordu University, Ordu, Türkiye

**Keywords:** Pediatric burns, Conservative treatment, Clinical outcomes

## Abstract

**Background:**

Burn injuries are a major cause of morbidity and mortality in children, particularly in low- and middle-income countries where access to specialized burn centers is limited. Advances in modern dressings have facilitated conservative management, but evidence from non-burn centers remains scarce.

**Methods:**

We retrospectively analyzed 520 pediatric patients with burn injuries involving ≤ 20% total body surface area (TBSA) treated between January 2021 and February 2025 in a tertiary hospital without a dedicated burn unit. All children were managed using a standardized conservative protocol incorporating silver-based, hyaluronic acid–based, alginogel, hydrogel, Tulle Gras, and hemoglobin spray dressings. Demographic features, etiology, TBSA, hospitalization, graft requirement, early complications, microbiologically confirmed infections, and mortality were evaluated. Long-term cosmetic and functional outcomes could not be systematically assessed and were therefore not primary endpoints of this study.

**Results:**

The median age was 4.2 years, and 65% of patients were aged 0–6 years. Scald burns accounted for 76.2% of injuries, and 65.0% of children had TBSA 1–10%. Overall, 98% of patients achieved acute wound closure without grafting, while 2% required split-thickness skin grafts, predominantly in burns > 15% TBSA or flame/electrical injuries. Keloid formation was documented in 5.0% and contractures in 0.4% of patients during early follow-up, but these rates likely underestimate true long-term scar prevalence due to incomplete late follow-up. Microbiologically confirmed infection occurred in 12.8% of hospitalized children, and no sepsis, invasive infections or mortality was observed.

**Conclusion:**

In pediatric patients with burns involving ≤20% TBSA, a structured conservative wound-care protocol can achieve high rates of acute wound closure with a very low need for grafting and no observed in-hospital mortality, even in a hospital without a dedicated burn center. These findings relate to acute outcomes; definitive long-term cosmetic and functional results could not be determined and warrant prospective studies with standardized long-term follow-up.

**Trial registration:**

Not applicable. This was a retrospective observational study that did not involve any prospective intervention or randomization.

## Background

Burn injuries remain a major global public health concern and are among the leading causes of accidental trauma and mortality in the pediatric population. According to the World Health Organization (WHO), more than 180,000 deaths occur each year due to burns, and a significant proportion of these fatalities involve children under the age of five [1]. Children are particularly vulnerable to severe burn injuries because of their limited motor coordination, underdeveloped hazard perception, and dependence on caregivers [2].

Beyond mortality, pediatric burns often result in long-term complications such as hypertrophic scarring, joint contractures, growth retardation, and profound psychosocial morbidity. These sequelae impose substantial economic and social burdens on families and healthcare systems alike [3]. In low- and middle-income countries, the limited availability of dedicated burn units and rehabilitation services further exacerbates these negative outcomes [4].

Over the past decade, advances in wound care technology have brought non-surgical, conservative approaches to the forefront of pediatric burn management. Silver-impregnated dressings provide broad antimicrobial coverage and support favorable wound conditions [5]. Hyaluronic acid (HA)–based preparations contribute to tissue repair by modulating inflammation and promoting granulation [6]. Enzymatic alginogel formulations facilitate gentle autolytic debridement and assist with exudate control [7]. Antiseptic Tulle Gras dressings, with their low-adherence structure, enable less traumatic dressing changes and remain a cost-effective option for superficial and partial-thickness injuries [8]. Hydrogel-based dressings offer a cooling effect and help maintain wound hydration [9]. Purified hemoglobin spray enhances topical oxygen availability, which has been associated with improved healing dynamics in recent clinical studies [10].

Collectively, these advanced topical therapies reduce the need for extensive surgical intervention and improve uncomplicated healing rates, particularly in children with ≤ 20% total body surface area (TBSA**)** involvement.

This study aims to describe the epidemiological characteristics and clinical outcomes of pediatric burn cases managed over four years using modern conservative treatment approaches in a tertiary hospital without a dedicated burn unit. The objective is not to present algorithmic or predictive “data-driven” decision models, but rather to evaluate outcomes associated with a structured conservative management protocol in this resource-variable setting.

## Materials and methods

### Study design and setting

This retrospective clinical study aimed to evaluate the epidemiological characteristics and treatment outcomes of pediatric burn patients managed with modern conservative methods. The study included children treated between January 2021 and February 2025 at Ordu University Training and Research Hospital, a tertiary referral center in Türkiye. All patients were managed in the Pediatric Surgery Outpatient Clinic and a dedicated burn treatment room. The study protocol was reviewed and approved by the Ordu University Clinical Research Ethics Committee (Approval No: 172, Date: 09.05.2025).

### Participants and burn classification

A total of 552 pediatric patients were initially screened for eligibility. Of these, 32 children were excluded due to incomplete documentation (*n* = 21) or major comorbidities that could potentially influence wound healing, including congenital heart disease, immunodeficiency, and metabolic disorders (*n* = 11). The final analysis therefore included 520 patients with complete demographic, clinical, and treatment data. Patients were categorized according to age group (0–6, 7–12, and 13–18 years) and burn etiology (scald from hot liquid or food, contact, flame, or electrical burns). The total body surface area (TBSA) was determined using the Lund–Browder chart and classified as TBSA 1–10% or 11–20%. No patient had TBSA exceeding 20%. Patients with incomplete data or major comorbidities were excluded.

### Treatment protocol

All patients were managed according to a standardized conservative wound care protocol routinely applied in our pediatric surgery unit. This protocol included antiseptic wound cleansing with active chlorine solutions, gentle mechanical removal of loose epidermal debris, and, when required, minimal enzymatic debridement. Dressing selection followed the principles of moist wound healing and was guided by clinical burn depth, exudate level, presence of blisters or slough, patient age and pain sensitivity, and product availability. Modern dressing materials used within this protocol included silver-based dressings (Atrauman Ag^®^), hyaluronic acid preparations (Hyalomatrix^®^), enzymatic alginogel (Flaminal^®^), antiseptic Tulle Gras dressings, hydrogel sheets, and purified hemoglobin spray (Granulox^®^). Dressing changes were typically performed every 2–3 days, with longer intervals in low-exudate superficial burns and more frequent assessments in cases with suspected colonization or increased exudate.

When clinically indicated, surgical procedures such as debridement, escharotomy, or escharectomy were performed. Split-thickness skin grafting was reserved for cases unresponsive to conservative therapy or when a full-thickness component was suspected. Fluid therapy and antibiotic regimens were individualized based on patient age, clinical presentation, and laboratory findings under pediatric supervision.

### Infection surveillance and antibiotic management

For all hospitalized patients, routine infection surveillance was performed according to the institutional pediatric burn care policy. Wound and/or blood cultures were obtained in cases with clinical suspicion of infection, including increased exudate, delayed epithelialization, fever, leukocytosis, or malodor. Empirical antibiotic therapy, when indicated, was initiated under the supervision of a pediatric infectious disease specialist and subsequently modified based on culture results and antimicrobial susceptibility patterns. Patients without clinical signs of infection did not receive prophylactic systemic antibiotics. All antimicrobial decisions, adjustments, and discontinuations were made by the supervising pediatricians in accordance with contemporary pediatric burn infection guidelines.

### Statistical analysis

All statistical analyses were performed using SPSS version 26.0 (IBM Corp., Armonk, NY, USA). Continuous variables were expressed as mean ± standard deviation (SD) or median with interquartile range (IQR), while categorical variables were presented as frequencies (n) and percentages (%).

Comparisons between subgroups defined by age, etiology, and TBSA were conducted using the Chi-square (χ²) or Fisher’s exact test for categorical data, and the independent-samples t-test or Mann–Whitney U test for continuous variables, as appropriate. For graft requirement and complication rates, relative risk (RR) with 95% confidence intervals (CIs) was calculated. A two-tailed p-value < 0.05 was considered statistically significant.

## Results

### Demographic and clinical characteristics

A total of 520 pediatric patients were included. The median age was 4.2 years (IQR 2.0–8.1), and 65% (*n* = 338) were in the 0–6-year group, 20% (*n* = 104) in the 7–12-year group, and 15% (*n* = 78) in the 13–18-year group.

There were 301 boys (57.8%) and 219 girls (42.2%), with a male-to-female ratio of 1.4:1 (Table 1).

**Table 1:** Demographic and clinical characteristics of pediatric burn patients (*n* = 520)

Variable	*n*	%
Age Groups		
0–6 years	338	65.0
7–12 years	104	20.0
13–18 years	78	15.0
Sex		
Male	301	57.8
Female	219	42.2
Burn Etiology		
Scald (hot water/food spill)	396	76.2
Contact burn	98	18.9
Flame burn	15	2.9
Electrical burn	10	2.0
Total Body Surface Area (TBSA)		
1–10%	338	65.0
11–20%	182	35.0
Hospitalization Status		
Inpatient	224	43.0
Outpatient follow-up	296	57.0
Mean Length of Stay (days)	8.5 ± 2.5	–

Values are presented as n (%) unless otherwise specified. Length of stay is provided as mean ± SD

### Burn etiology and TBSA distribution

The leading cause of injury was scald burns from hot liquids or food, affecting 396 patients (76.2%). Contact burns accounted for 18.9% of cases, followed by flame burns (2.9%) and electrical burns (2.0%). Among the 15 flame-burn cases, most injuries were clinically categorized as mixed superficial and partial-thickness flash burns, and only five children were suspected to have full-thickness components. All electrical injuries (*n* = 10) resulted from low-voltage exposure (< 1000 V); no high-voltage electrical burns were identified in this cohort.

Most patients (338, 65.0%; 95% CI: 60.7–69.0) had TBSA 1–10%, while 182 children (35.0%; 95% CI: 31.0–39.3) had TBSA 11–20%. No patient had TBSA exceeding 20%. The mean TBSA was 8.4% ± 4.1%, with a median of 7% (range 1–20%). In the 11–20% subgroup, the median TBSA was 15% (IQR 13–18%).

Hospitalization was significantly more frequent among patients with TBSA > 10% compared to those with ≤ 10% (56.6% vs. 35.2%; *p* < 0.001).

### Treatment modalities and outcomes

Overall, 224 patients (43%) were hospitalized, while 296 (57%) were treated on an outpatient basis.

**Fig. 1:**
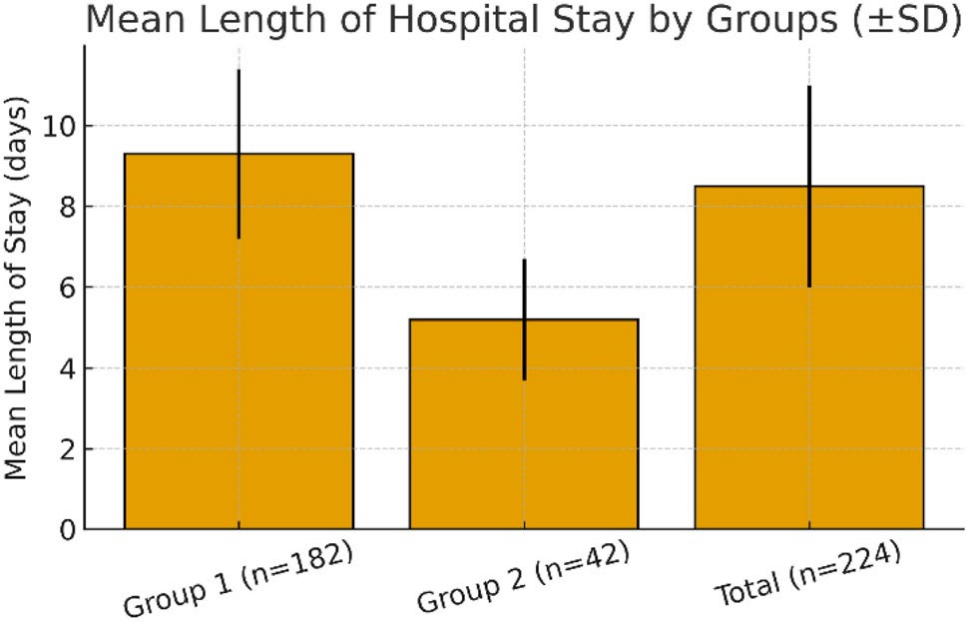
Comparison of the mean hospital stay duration between study groups. Group 1 (n=182) represents patients with TBSA >10%, while Group 2 (n=42) includes those with TBSA ≤10%. The mean length of stay was significantly longer in Group 1 (9.3 ± 2.1 days) compared to Group 2 (5.2 ± 1.5 days, p < 0.001). The overall mean hospital stay for all inpatients was 8.5 ± 2.5 days

The mean hospital stay was 8.5 ± 2.5 days, significantly longer for patients with TBSA > 10% (9.3 ± 2.1 vs. 5.2 ± 1.5 days; *p* < 0.001) (Table 1; Fig. 1).

**Fig. 2:**
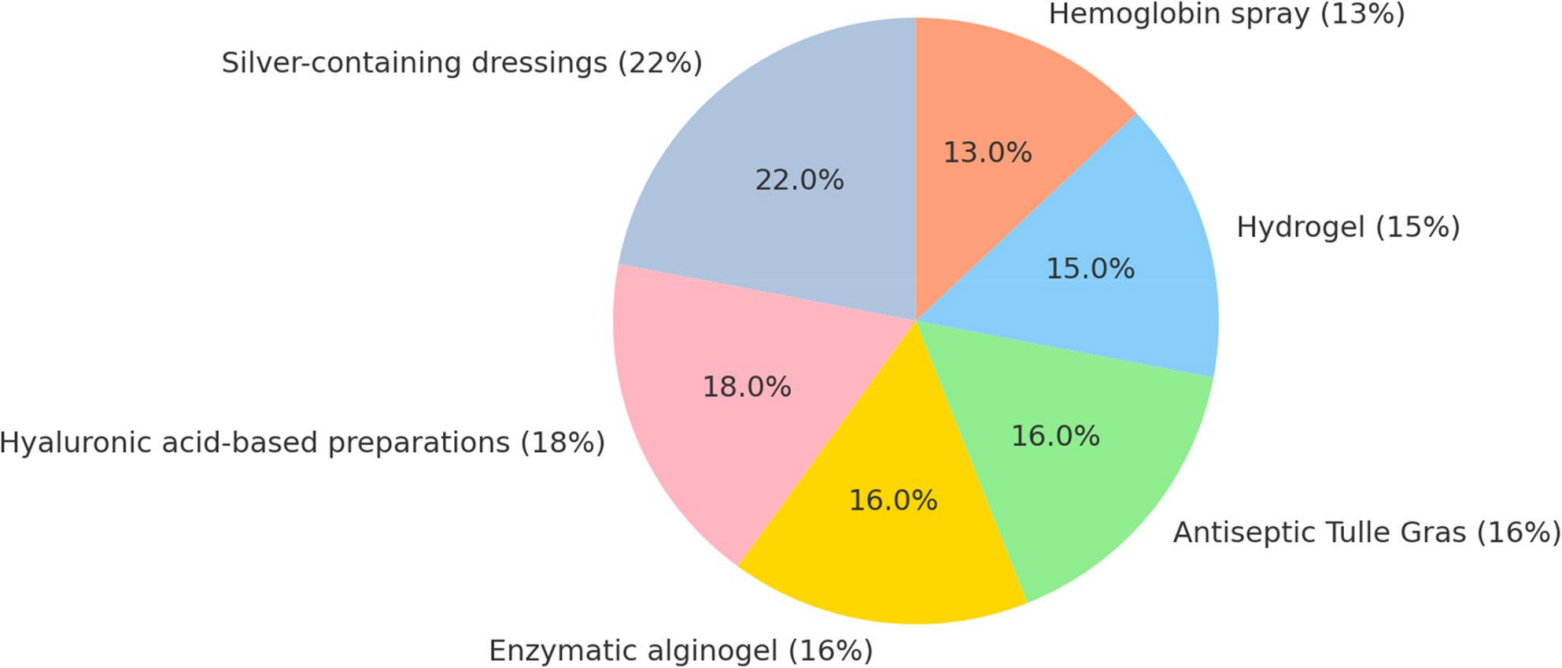
Distribution of modern wound care products used in the study. Among the pediatric burn patients, silver-containing dressings were used in 22% of cases, followed by hyaluronic acid-based preparations (18%), enzymatic alginogel (16%), antiseptic Tulle Gras (16%), hydrogel (15%), and hemoglobin spray (13%). In some cases, multiple products were applied concurrently. Overall, 98% of patients achieved complete recovery with conservative management, highlighting the effectiveness of these modern wound care approaches

Among modern wound care modalities, silver-based dressings were used in 22%, hyaluronic acid preparations in 18%, enzymatic alginogel in 16%, antiseptic Tulle Gras dressings in 16%, hydrogel in 15%, and hemoglobin spray in 13% of cases (Fig. 2). These dressing modalities were selected according to the standardized conservative wound care protocol described in the Methods section.

With these combinations, 98% of patients achieved complete healing without grafting, while only 10 patients (2%) required split-thickness skin grafts. All grafted patients had TBSA involvement greater than 15% or sustained flame/electrical burns. Among the flame-burn subgroup specifically, four children required grafting, each with TBSA > 15%.

The need for grafting was significantly higher among patients with TBSA > 15% (RR = 8.5; 95% CI: 3.2–22.3; *p* < 0.01).

### Complications and mortality

Keloid formation was observed in 26 patients (5.0%; 95% CI: 3.3–7.2) more frequently in those with TBSA > 10% (*p* = 0.04). None of the 26 children who developed keloids had undergone grafting. Both patients who developed contractures had superficial-to-deep partial-thickness scald burns and neither had been grafted; both complications occurred despite complete healing without surgery.

Only two patients (0.4%) developed contractures, successfully managed with physical therapy (95% CI: 0.05–1.5). No mortality occurred during the study period (0%; 95% CI: 0–0.7) (Table 2).

**Table 2:** Clinical outcomes, infection profile, and complications in pediatric burn patients

Variable	*n*	%
Acute Wound Outcomes		
Acute wound closure without surgery	510	98.0
Split-thickness skin graft	10	2.0
Infection and Microbiology		
Cultures obtained (hospitalized patients)	218	97.3
Culture-positive infections	28	12.8
Complications		
Local keloid formation	26	5.0
Contracture	2	0.4
Mortality	0	0.0

**Fig. 3:**
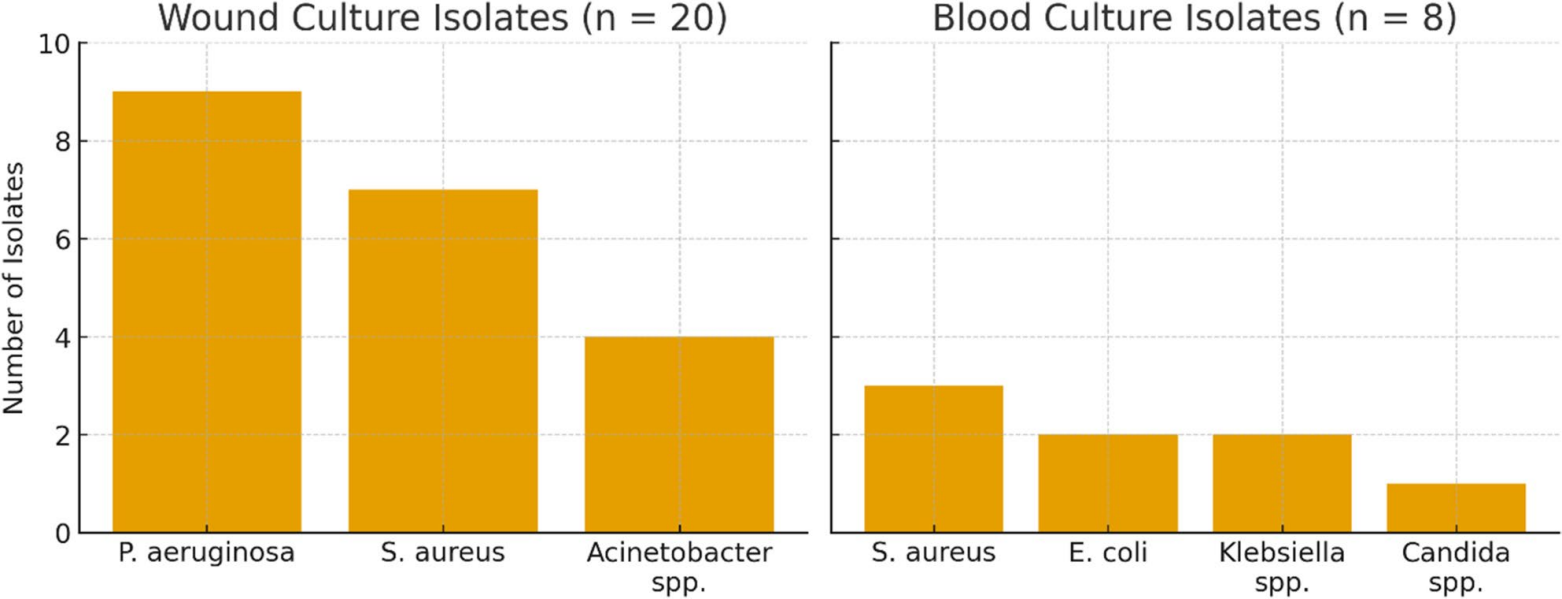
Distribution of wound and blood culture isolates in pediatric burn patients. Figure 3 illustrates the distribution of microorganisms isolated from positive wound and blood cultures among hospitalized pediatric burn patients (total culture-positive cases = 28). Among wound cultures (n = 20), Pseudomonas aeruginosa was the most frequently isolated organism (n = 9, 45%), followed by Staphylococcus aureus (n = 7, 35%) and Acinetobacter spp. (n = 4, 20%). Among blood cultures (n = 8), Staphylococcus aureus (n = 3), Escherichia coli (n = 2), Klebsiella spp. (n = 2), and Candida spp. (n = 1) were identified. Notably, none of the culture-positive children met clinical criteria for sepsis, consistent with pediatric burn literature indicating that localized colonization is common, while progression to invasive infection remains uncommon

Among the 224 hospitalized patients, wound or blood cultures were obtained from 218 children (97.3%). A total of 28 patients had microbiologically confirmed infection (12.8%; 95% CI: 8.7–18.0). Wound cultures most frequently yielded *Pseudomonas aeruginosa*, *Staphylococcus aureus*, and *Acinetobacter* spp., whereas blood cultures mainly grew *Staphylococcus aureus*, *Escherichia coli*, *Klebsiella* spp., and *Candida* species (Table 2; Fig. 3). No cases of sepsis or culture-proven invasive fungal infection were identified.

## Discussion

In this study, the predominance of the 0–6-year age group (65%) among pediatric burn patients aligns with previous reports emphasizing that younger children are at a higher risk of burn injuries. Consistent with earlier studies from developing countries, domestic scald injuries were the most common cause of burns, highlighting the household as the primary setting for pediatric burn accidents [1–4]. In our series, scald burns accounted for 76.2% of all injuries, consistent with global pediatric burn epidemiology.

A major component of our treatment approach was the use of modern conservative wound care modalities, often in combination. Silver-based dressings (22%), hyaluronic acid preparations (18%), enzymatic alginogel (16%), antiseptic Tulle Gras dressings (16%), hydrogel sheets (15%), and hemoglobin spray (13%) were the most frequently applied. This multimodal strategy achieved complete healing without grafting in 98% of patients, closely corresponding to the 2–4% graft requirement reported by Ball et al. in partial-thickness burns involving < 10% TBSA [11]. Likewise, Mistry et al. found that long-term scar outcomes were comparable between conservative and surgical approaches, reinforcing that appropriate wound management can minimize the need for operative intervention [12].

Although the Chinese Burn Association (CBA) 2023 consensus recommends early excision and grafting for confirmed deep partial-thickness burns, these recommendations are largely based on centers with access to objective depth-assessment tools such as Laser Doppler Imaging (LDI) and predominantly adult data [13]. In our setting, burn depth was evaluated clinically, and in children—who have greater regenerative capacity than adults—many injuries clinically suspected to be deep partial-thickness healed successfully with conservative management.

This does not contradict the CBA principles; rather, it highlights that the optimal management strategy may differ in pediatric patients and in centers without LDI, where a “selective, carefully monitored conservative-first approach” may prevent unnecessary surgery while still allowing timely intervention for true deep burns. Similar findings have been documented in Dutch and Scandinavian pediatric burn cohorts, where conservative monitoring of clinically borderline burns often leads to complete re-epithelialization [14, 15].

An additional advantage of modern conservative management was the reduced need for frequent dressing changes. Many patients required dressing replacement only every 2–3 days, and in some cases up to a week, which decreased pain, reduced sedation/anesthesia requirements, and minimized fasting periods associated with procedural sedation. This aligns with Greenhalgh’s observation that “pain-free, minimally invasive wound care improves recovery in pediatric partial-thickness burns.” [16].

The low incidence of keloid and contracture formation (5% and 0.4%, respectively) may reflect the benefits of early wound management, appropriate dressing selection, and structured follow-up. Previous studies have reported higher hypertrophic scar rates (17–30%), especially in cases with healing times exceeding 21 days [17]. The relatively low TBSA involvement (≤ 20%) and likely shorter healing duration in our cohort may explain the favorable outcomes. However, it is important to note that these findings represent early follow-up outcomes; hypertrophic scars and keloids typically become clinically apparent 3–6 months after injury. Because long-term follow-up was incomplete for many patients, the true prevalence of late-onset scarring may be higher than reported. Accordingly, our conclusions emphasize acute wound outcomes rather than definitive long-term scar rates, highlighting the need for future studies with standardized long-term scar assessment.

The microbiologically confirmed infection rate in our hospitalized cohort was 12.8%, which is lower than the 14–38% infection rates reported in several pediatric burn studies, including those managed with predominantly conservative approaches [18, 19]. Consistent with prior reports, *Pseudomonas aeruginosa* and *Staphylococcus aureus* were the most common wound isolates, while bloodstream infections were mainly associated with *Staphylococcus aureus*, *Escherichia coli*, *Klebsiella* spp., and *Candida* species. Notably, no sepsis or invasive fungal infection occurred, supporting the safety of our conservative protocol in children with ≤ 20% TBSA involvement.

Several limitations should be acknowledged. First, the retrospective design inherently limited the completeness and standardization of the dataset, particularly regarding healing time and detailed documentation of colonization dynamics. Although microbiological sampling was performed in nearly all hospitalized patients, the timing of cultures and the absence of standardized colonization surveillance restrict interpretation of infection trajectories. Second, burn depth was assessed clinically in the absence of objective tools such as Laser Doppler Imaging (LDI), which may have led to misclassification of some deep partial-thickness injuries. Third, long-term cosmetic, functional, and psychosocial outcomes were not evaluated. Given that hypertrophic scars and keloids often develop months after re-epithelialization, follow-up beyond the acute healing phase was incomplete, and thus the reported low rates of keloid and contracture likely underestimate true long-term scar incidence. Future multicenter studies should incorporate structured follow-up extending to at least 6–12 months after injury.

Our description of treatment “success” refers specifically to acute wound closure and avoidance of surgery, rather than long-term cosmetic or functional outcomes, which could not be fully evaluated due to the limitations outlined above.

Regarding costs, although advanced dressing products are more expensive, reduced dressing frequency, lower sedation/anesthesia requirements, and avoidance of surgery may offset part of the financial burden. Still, affordability remains a significant challenge in low- and middle-income countries, underscoring the need for future studies to evaluate simpler and more cost-effective dressing alternatives within structured conservative care pathways.

## Conclusion

Our findings suggest that in pediatric patients with TBSA ≤ 20%, modern conservative wound management can achieve high rates of acute wound closure with very limited need for surgical intervention and no observed mortality. These results support the practicality and safety of conservative burn care in hospitals without dedicated burn centers, particularly in resource-limited settings. Additionally, community education, timely presentation, and improved training of healthcare personnel remain important components for optimizing pediatric burn care.

## Data Availability

The datasets generated and/or analyzed during the current study are not publicly available due to institutional privacy restrictions but are available from the corresponding author on reasonable request.

## Abbreviations

TBSA: Total Body Surface Area
WHO: World Health Organization
HA: Hyaluronic Acid
RR: Relative Risk
CI: Confidence Interval
IQR: Interquartile Range
SD: Standard Deviation
SPSS: Statistical Package for the Social Sciences
Ag: Silver (from silver-based dressings)
CBA: Chinese Burn Association
LDI: Laser Doppler Imaging
